# Sinularin from Indigenous Soft Coral Attenuates Nociceptive Responses and Spinal Neuroinflammation in Carrageenan-Induced Inflammatory Rat Model

**DOI:** 10.3390/md10091899

**Published:** 2012-08-24

**Authors:** Shi-Ying Huang, Nan-Fu Chen, Wu-Fu Chen, Han-Chun Hung, Hsin-Pai Lee, Yen-You Lin, Hui-Min Wang, Ping-Jyun Sung, Jyh-Horng Sheu, Zhi-Hong Wen

**Affiliations:** 1 Department of Marine Biotechnology and Resources, Asia-Pacific Ocean Research Center, National Sun Yat-sen University, Kaohsiung 80424, Taiwan; Email: johnjohnkings@gmail.com (S.-Y.H.); chas6119@gmail.com (Y.-Y.L.); sheu@mail.nsysu.edu.tw (J.-H.S.); 2 Division of Neurosurgery, Department of Surgery, Kaohsiung Armed Forces General Hospital, Kaohsiung 80284, Taiwan; Email: chen06688@gmail.com; 3 Department of Neurosurgery, Kaohsiung Chang Gung Memorial Hospital and Chang Gung University College of Medicine, Kaohsiung 83301, Taiwan; Email: ma4949@adm.cgmh.org.tw; 4 Doctoral Degree Program in Marine Biotechnology, National Sun Yat-sen University, Kaohsiung 80424, Taiwan; Email: hanchun25@gmail.com; 5 Sections of Orthopedic Surgery, Pingtung Christian Hospital, #60 Da-Lan Road, Pingtung 900, Taiwan; Email: hplee0929@gmail.com; 6 Department of Fragrance and Cosmetic Science, Center of Excellence for Environmental Medicine, Kaohsiung Medical University, Kaohsiung 80708, Taiwan; Email: davidw@kmu.edu.tw; 7 Taiwan Coral Research Center, National Museum of Marine Biology and Aquarium, Pingtung 944, Taiwan; Email: pjsung@nmmba.gov.tw

**Keywords:** sinularin, carrageenan, inflammatory pain, spinal neuroinflammation, transforming growth factor-β1, natural marine compound

## Abstract

Three decades ago, the marine-derived compound sinularin was shown to have anti-edematous effects on paw edema induced by carrageenan or adjuvant. To the best of our knowledge, no new studies were conducted to explore the bioactivity of sinularin until we reported the analgesic properties of sinularin based on *in vivo* experiments. In the present study, we found that sinularin significantly inhibits the upregulation of proinflammatory proteins, inducible nitric oxide synthase (iNOS), and cyclooxygenase-2 (COX-2) and upregulates the production of transforming growth factor-β (TGF-β) in lipopolysaccharide (LPS)-stimulated murine macrophage RAW 264.7 cells according to western blot analysis. We found that subcutaneous (s.c.) administration of sinularin (80 mg/kg) 1 h before carrageenan injection significantly inhibited carrageenan-induced nociceptive behaviors, including thermal hyperalgesia, mechanical allodynia, cold allodynia, and hindpaw weight-bearing deficits. Further, s.c. sinularin (80 mg/kg) significantly inhibited carrageenan-induced microglial and astrocyte activation as well as upregulation of iNOS in the dorsal horn of the lumbar spinal cord. Moreover, s.c. sinularin (80 mg/kg) inhibited carrageenan-induced tissue inflammatory responses, redness and edema of the paw, and leukocyte infiltration. The results of immunohistochemical studies indicate that s.c. sinularin (80 mg/kg) could upregulate production of TGF-β1 in carrageenan-induced inflamed paw tissue. The present results demonstrate that systemic sinularin exerts analgesic effects at the behavioral and spinal levels, which are associated with both inhibition of leukocyte infiltration and upregulation of TGF-β1.

## 1. Introduction

**Figure 1:**
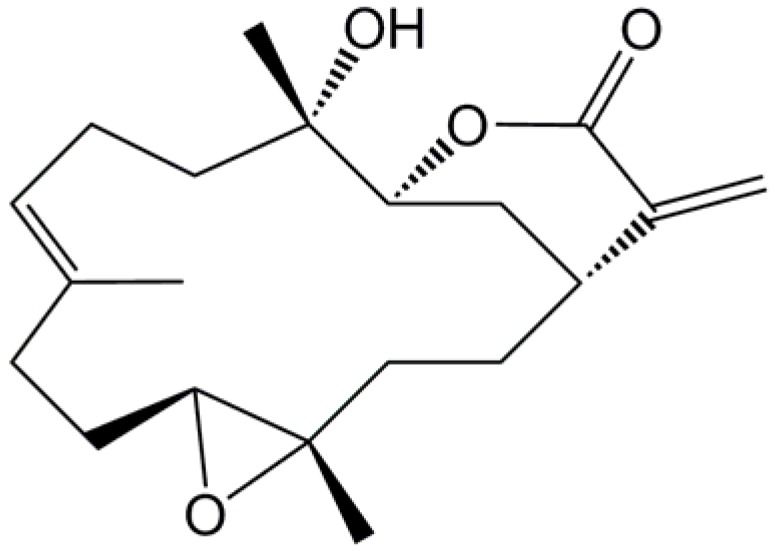
Chemical structure of sinularin (5,15-dioxatricyclo[12.3.1.0(4,6)]octadec-9-en-16-one).

Marine-derived compounds obtained from soft corals are believed to yield many potential candidate compounds for treating inflammatory diseases, particularly for treating pain [1,2,3,4]. The natural marine compound sinularin (Figure 1) has been well-studied and was shown by Weinheimer *et al*. in 1977 to have anticancer activity against the human epidermoid carcinoma cell line (KB) and the murine P388 lymphocytic leukemia cell line (PS) cell line from the soft coral *Sinularia flexibilis* [5]. In 1978, Kazlauskas *et al*. isolated sinularin from the same soft coral, but referred to the compound as flexibilide [6]. Later, in 1980, Buckle *et al*. reported that oral administration of sinularin reduced carrageenan-induced paw edema 3 h after carrageenan injection and that chronic oral sinularin inhibited adjuvant-induced paw swelling (periarthritis model) over a 21-day period after adjuvant administration [7]. After Buckle *et al*. used rat models to show the anti-inflammatory activity of sinularin [7], scientists explored other bioactivities of sinularin, such as its cardiovascular [8] and antimicrobial [9] activities. Sinularin was originally isolated from the soft coral *Sinularia flexibilis* collected from Hayman Island on the Great Barrier Reef of Australia [5]. Using an *in vitro* anti-inflammatory assay system, we found that sinularin, isolated from the same genus but from a different species of soft coral, *Sinularia querciformis* collected from Dongsha Islands off Taiwan, significantly inhibited upregulation of proinflammatory proteins, particularly inducible nitric oxide synthase (iNOS) and cyclooxygenase-2 (COX-2), in lipopolysaccharide (LPS)-stimulated murine macrophage RAW 264.7 cells. However, there are two important points regarding the bioactivity of sinularin that remain unclear. First, no previous studies have explored the analgesic activity of sinularin. Second, potential mechanisms of sinularin’s anti-inflammatory activity remain unclear.

Inhibiting iNOS and COX-2 expression in LPS-treated RAW 264.7 macrophages can be used as an *in vitro* index for screening compounds with anti-inflammatory activity; we previously succeeded in screening compounds from natural marine compounds using this *in vitro* system [1,4,10,11]. Using this *in vitro* system, we found that sinularin is a potential anti-inflammatory compound that has the ability to prevent iNOS and COX-2 upregulation. Systemic iNOS and COX-2 reportedly play key roles in inflammatory processes and in pain response. Expression of the iNOS and COX-2 proteins was in carrageenan-induced inflamed rat paws; additionally, iNOS and COX-2 protein expression was not detected in the paw tissue of naïve rats [1,12]. Systemic injection of an iNOS-selective inhibitor (*N*-iminoethyl-l-lysine and aminoguanidine) or a COX-2-selective inhibitor (SC-58125) inhibited carrageenan-induced paw edema [12,13]. Furthermore, carrageenan-induced thermal hyperalgesia can be reduced through systemic administration of the iNOS selective inhibitor ONO-1714 or the COX-2 selective inhibitor SC-58125 [13,14]. Therefore, we focused on whether a combination of systemic sinularin and inhibiting the induction of iNOS and COX-2 *in vitro* has an antinociceptive and anti-inflammatory effect on carrageenan-induced nociceptive behaviors (thermal hyperalgesia, mechanical allodynia, cold allodynia, and hindpaw weight-bearing deficits) and histological changes. Spinal microglial and astrocyte activation are known to play important roles in nociceptive sensitization [2,3,15,16,17,18,19,20,21,22]. Many studies have shown that compounds exert their analgesic effects by inhibiting microglial and astrocyte activation [2,3,21,22]. However, no studies have been conducted to examine the ability of sinularin to inhibit spinal microglial and astrocyte activation. In the present study, we examined whether sinularin affects spinal microglial and astrocyte activation accompanied by nociceptive behaviors and elucidate possible anti-inflammatory mechanisms in a carrageenan-injected rat model. Additionally, we also examined the effect of sinularin on transforming growth factor-β (TGF-β) under inflammatory conditions.

## 2. Results

### 2.1. Effect of Sinularin on LPS-Induced iNOS, COX-2, and TGF-β Protein Expression in Macrophages

**Figure 2:**
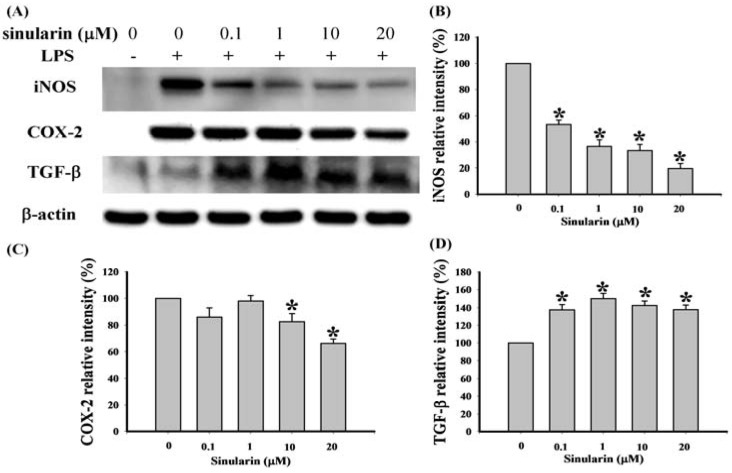
Effect of sinularin on protein expression of pro-inflammatory inducible nitric oxide synthase (iNOS) and cyclooxygenase-2 (COX-2) and anti-inflammatory transforming growth factor-β (TGF-β) in RAW 264.7 cells induced by lipopolysaccharide (LPS; 0.01 μg/mL). (**A**) Western blots of iNOS, COX-2, TGF-β, and β-actin proteins from RAW 264.7 cells; (**B**) relative density of immunoblot of iNOS; (**C**) relative density of immunoblot of COX-2; (**D**) relative density of immunoblot of TGF-β. Relative intensity of the LPS-stimulated group was defined as 100%. Band intensities were quantified using densitometry and are indicated as the percentage change relative to that of the LPS-stimulated group. Western blotting using β-actin was performed to verify loading of equivalent amounts of protein in each lane. This experiment was repeated 3 times. * *P *< 0.05 compared with the LPS-stimulated group.

We used western blot analysis to evaluate expression of pro-inflammatory proteins (iNOS and COX-2) and TGF-β in the LPS-stimulated RAW 264.7 macrophage cells using whole cell lysates and 3 antibodies against mouse macrophage iNOS, COX-2, and TGF-β, respectively (Figure 2). Dose responses of sinularin for inhibition of LPS-induced 130-kDa iNOS and 71-kDa COX-2 and up-regulation of LPS-induced 13-kDa TGF-β are shown in Figure 2. At 0.1, 1, 10, and 20 μM doses of sinularin, the levels of iNOS protein were significantly reduced to 53.45 ± 3.27%, 36.45 ± 5.15%, 33.38 ± 4.61%, and 19.48 ± 3.95% of the control level, respectively (Figure 2B). At concentrations of 10 and 20 μM doses of sinularin, the level of COX-2 protein were also reduced significantly to 82.72 ± 6.17% and 66.23 ± 3.27% of the control level, respectively (Figure 2C. At 0.1, 1, 10, and 20 μM doses of sinularin, the level of TGF-β protein were increased significantly to 137.75 ± 5.97%, 149.82 ± 6.15%, 142.71 ± 4.57%, and 138.02 ± 5.15% of the control level, respectively (Figure 2D). Therefore, three changes in protein levels take place at 10 μM and 20 μM doses of sinularin, including inhibition of iNOS and of COX-2 as well as upregulation of TGF-β.

**Figure 3:**
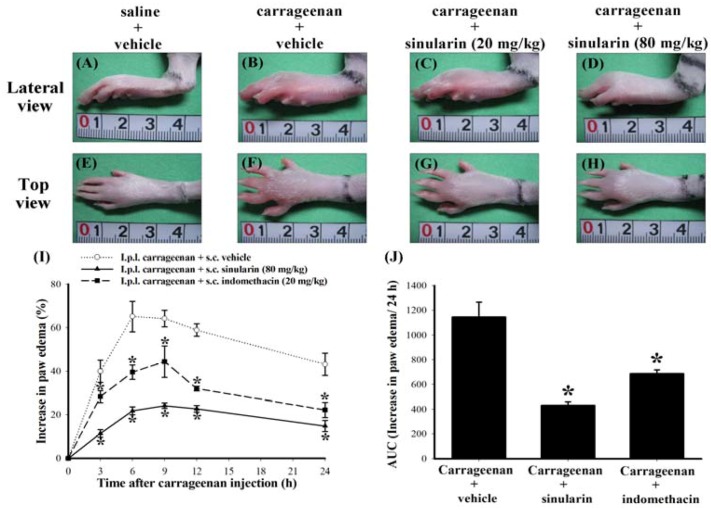
Anti-redness and anti-edematous effects of subcutaneous (s.c.) sinularin in intraplantar (i.p.l.) carrageenan-induced paw edema model. Photographic images show the gross pathology of paws from i.p.l. saline plus s.c. vehicle (dimethyl sulfoxide [DMSO]) (**A**, **E**), i.p.l. carrageenan plus s.c. vehicle (**B**, **F**), i.p.l. carrageenan plus s.c. sinularin (20 mg/kg) (**C**, **G**), and i.p.l. carrageenan plus s.c. sinularin (80 mg/kg) (**D**, **H**) groups. The lateral view of paws are shown in A, B, C, and D; top view of paws are shown in E, F, G, and H. The images (A–H) were taken 6 h after i.p.l. saline or carrageenan injection (as well as 7 h after s.c. vehicle or sinularin injection). The unit of scale bars shown in images A–H is in centimeter. S.c. sinularin of 20 mg/kg and 80 mg/kg reduced levels of carrageenan-induced edema of paw, and s.c. sinularin 80 mg/kg also clearly inhibited redness induced by carrageenan in rats than that by 20 mg/kg sinularin. We used 20 mg/kg indomethacin as a positive control. The time course of the percentage change of the increase in paw volume induced by i.p.l. injection of carrageenan in rats pretreated with vehicle, sinularin (80 mg/kg), or indomethacin (20 mg/kg) (**I**), administrated 1 h before carrageenan injection. Basal volume of each rat paw before i.p.l. saline or carrageenan injection was considered to be 100%, and the increase in paw volume is shown as the percentage change from the basal values by subtracting the basal paw volume from the paw volume measured at each time point. Area under the edematous effect-time curve (**J**), which was transformed from image I, for s.c. vehicle, sinularin (80 mg/kg), or indomethacin (20 mg/kg) injection, extending from 3 to 24 h after i.p.l. carrageenan injection. Carrageenan-induced paw edema was well-developed by nearly 3 h and persisted for at least 24 h. This phenomenon was significantly reduced by s.c. sinularin (80 mg/kg) or indomethacin (20 mg/kg). The duration of the anti-edematous effects of sinularin (80 mg/kg) or indomethacin (20 mg/kg) are shown as the area under the curve (AUC). Each point or bar in images I and J represent the mean ± standard error of the mean (SEM) of 6 rats per group. * *P *< 0.05 compared with the same time points in i.p.l. carrageenan plus s.c. vehicle group.

### 2.2. Effect of Systemic Sinularin Injection on Carrageenan-Induced Redness and Edema of the Paw

According to the results in LPS-stimulated RAW 264.7 macrophages, we considered that 10–20 μM doses of sinularin both inhibit LPS-induced iNOS and COX-2 and upregulates LPS-induced TGF-β. To estimate an effective *in vivo* dosage of sinularin, we used a previous formula developed by Caraci *et al*. to calculate *in vivo* dosage for central administration based on *in vitro* dosage [23], considering that the CSF volume of a 300 g rat equals about 580 μL [24]. Hence, we estimated that the dosage of sinularin for central administration to a 300 g rat should be approximately 1.94–3.87 μg. Systemic dosage (mg/kg level) is well-known to be approximately equivalent to 1000 times of central dosage (μg/kg level), so we supposed that the sinularin dosage for systemic administration to a rat was at least about 6.47–12.9 mg/kg. Therefore, we selected two dosages of sinularin, 20 mg/kg and 80 mg/kg, administrated 1 h before carrageenan injection, for following preliminary tests. We prepared the following 4 groups of rats: intraplantar (i.p.l.) saline plus subcutaneous (s.c.) vehicle (dimethyl sulfoxide [DMSO]), i.p.l. carrageenan plus s.c. vehicle, i.p.l. carrageenan plus s.c. sinularin (20 mg/kg), and i.p.l. carrageenan plus s.c. sinularin (80 mg/kg) groups. At 6 h after i.p.l. carrageenan injection, carrageenan caused visible redness and swelling of rat paw (Figure 3B,F) compared with the i.p.l. saline plus s.c. vehicle group ( 3A,E B,F), both s.c. 20 mg/kg ( 3C,G D,H) sinularin inhibited carrageenan-induced paw edema; moreover, s.c. 80 mg/kg sinularin also clearly reduced redness induced by carrageenan in rats that received 20 mg/kg of sinularin. Next, we chose a dose of 80 mg/kg for evaluating the anti-edematous effect of sinularin on i.p.l. carrageenan-induced paw edema. We used 20 mg/kg indomethacin as a positive control [25,26]. The time course of percentage change of increased paw volume induced by i.p.l. injection of carrageenan was inhibited by sinularin (80 mg/kg) or indomethacin (20 mg/kg) (Figure 3I). Analysis of AUC of the edematous effect-time curve also supports the anti-edematous effect of sinularin (Figure 3J). Based on the above findings, we selected a dose of 80 mg/kg of sinularin for following nociceptive behavioral testing.

### 2.3. Effect of Systemic Injection of Sinularin on the Carrageenan-Induced Nociceptive Behaviors of Inflammatory Pain

**Figure 4:**
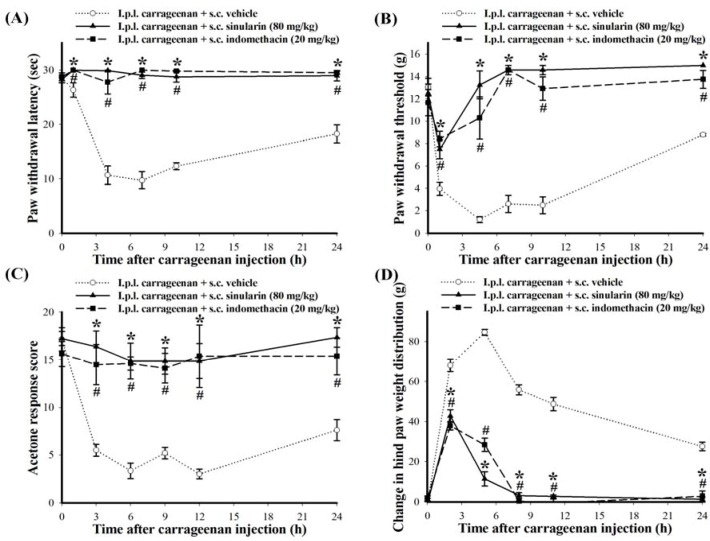
Time-courses of analgesic effects of subcutaneous (s.c.) sinularin (80 mg/kg) or indomethacin (20 mg/kg) on intraplantar (i.p.l.) carrageenan-induced nociceptive behaviors, including thermal hyperalgesia (**A**) mechanical allodynia; (**B**) cold allodynia; (**C**) and hindpaw weight-bearing deficits; (**D**) We used 20 mg/kg indomethacin as a positive control. Carrageenan-induced nociceptive behaviors peaked at 3–6 h and persisted for at least 24 h. This phenomenon was significantly inhibited by s.c. sinularin (80 mg/kg) or indomethacin (20 mg/kg). Each point in all figures represents the mean ± standard error of the mean (SEM) of 6 rats per group. * *P *< 0.05 between i.p.l. carrageenan plus s.c. sinularin (80 mg/kg) group compared with the same time points in i.p.l. carrageenan plus s.c. vehicle (dimethyl sulfoxide [DMSO]) group; ^#^ *P* < 0.05 between i.p.l. carrageenan plus s.c. indomethacin (20 mg/kg) group compared with the same time points in i.p.l. carrageenan plus s.c. vehicle group.

No significant differences in the baselines were observed for paw withdrawal latency (PWL), paw withdrawal threshold (PWT), acetone response score, or change in hind paw weight distribution among the experimental groups before carrageenan injection. Average baselines for PWL, PWT, acetone response score, and changes in hind paw weight distribution were 28.59 ± 0.45 s (*n* = 18), 12.50 ± 0.52 g (*n* = 18), 16.75 ± 0.66 points (*n* = 18), and −0.29 ± 1.10 g (*n* = 18), respectively. As expected, thermal hyperalgesia (PWL = 10.69 ± 1.72 s; *n* = 6), mechanical allodynia (PWT = 1.20 ± 0.26 g; *n* = 6), cold allodynia (acetone response score = 3.33 ± 0.79 points; *n* = 6), and hindpaw weight-bearing deficits (change in hind paw weight distribution = 84.63 ± 1.50 g; *n* = 6) of the carrageenan-injected paw peaked at 3–6 h after i.p.l. carrageenan injection; these nociceptive behaviors of inflammatory pain persisted for at least 24 h (Figure 4). Figure 4 presents the time course of analgesic effects of s.c. 80 mg/kg sinularin: anti-thermal hyperalgesia (Figure 4A, anti-mechanical allodynia (Figure 4B), anti-cold allodynia (Figure 4C, and anti-weight-bearing deficits (Figure 4D), respectively. Sinularin groups exhibited extended antinociceptive effects until at least 24 h after i.p.l. carrageenan injection, while s.c. injection of vehicle (100% DMSO) did not affect carrageenan-induced nociceptive behaviors. Additionally, s.c. indomethacin (20 mg/kg) also significantly inhibited these i.p.l. carrageenan-induced nociceptive behaviors until at least 24 h after i.p.l. carrageenan injection. In the present study, carrageenan plus s.c. sinularin-treated rats (20 and 80 mg/kg) did not exhibit any obvious side effects regarding external behavior, including locomotor function, during treatment. We then focused on the period of 24 h after s.c. 80 mg/kg of sinularin to determine whether inhibition of spinal neuroinflammatory processes are consistent with the antinociceptive effects of sinularin based on two primary considerations. First, these four i.p.l. carrageenan-induced nociceptive behaviors continued to 24 h after carrageenan injection. Second, s.c. administration of 80 mg/kg sinularin at 24 h after carrageenan injection retained antinociceptive efficacy.

**Figure 5:**
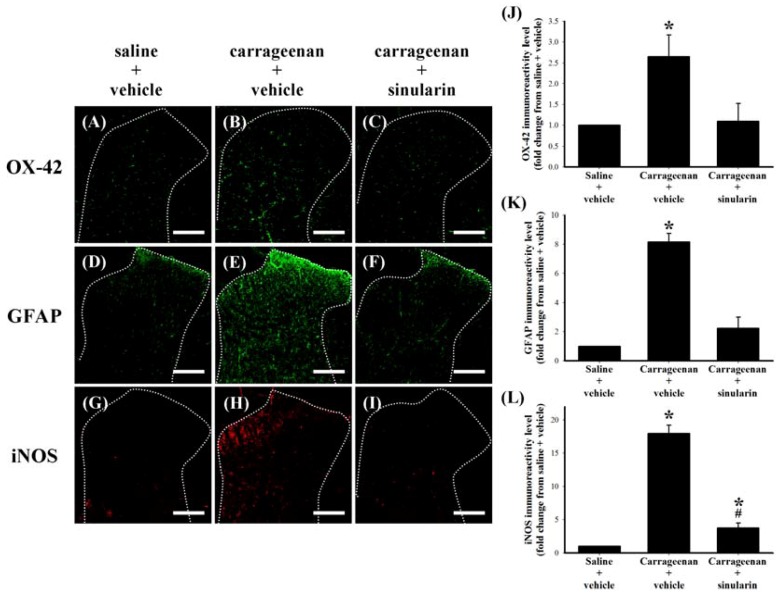
Inhibitive effect of subcutaneous (s.c.) sinularin on intraplantar (i.p.l.) carrageenan-induced spinal neuroinflammatory markers in the dorsal horn of the lumbar spinal cord. These spinal cord sections (10 μm) at 24 h after i.p.l. saline or carrageenan injection were from i.p.l. saline plus s.c. vehicle (dimethyl sulfoxide [DMSO]) (**A**, **D**, **G**), i.p.l. carrageenan plus s.c. vehicle (**B**, **E**, **H**), and i.p.l. carrageenan plus s.c. sinularin (80 mg/kg) (**C**, **F**, **I**) groups. Immunostaining images show cells labeled with OX-42 (green) (**A**–**C**), glial fibrillary acidic protein (GFAP) (green) (**D**–**F**), and iNOS (red) (**G**–**I**) in the spinal cord. Quantification of OX-42 (**J**), GFAP (**K**), and iNOS (**L**) immunoreactivity on the ipsilateral dorsal horn of the lumbar spinal gray matter, and each bar in images J–L represents the mean ± standard error of the mean (SEM) of 6 rats per group. S.c. sinularin significantly inhibited carrageenan-induced upregulation of spinal OX-42, GFAP, and iNOS immunoreactivity. Scale bars: 200 μm for all images (A–I). * *P *< 0.05 compared with the i.p.l. saline plus s.c. vehicle group; ^#^ *P* < 0.05 compared with the i.p.l. carrageenan plus s.c. vehicle group.

### 2.4. Effect of Systemic Sinularin Injection on Carrageenan-Induced Spinal Nociceptive Sensitization

Spinal tissue was collected at 24 h after carrageenan injection from the lumbar enlargement (L2–L4) for a spinal immunohistofluorescence assay of neuroinflammatory markers. OX-42-, GFAP-, or iNOS-immunoreactive cells were scattered throughout the ipsilateral dorsal horn of the lumbar spinal gray matter of i.p.l. saline plus s.c. vehicle (Figure 5A,D,G), i.p.l. carrageenan plus s.c. vehicle (Figure 5B,E,H), and i.p.l. carrageenan plus s.c. sinularin (80 mg/kg) (Figure 5C,F,I) groups. Compared with i.p.l. saline plus s.c. vehicle group, the immunoreactivity of OX-42 (Figure 5B), GFAP (Figure 5E), and iNOS (Figure 5H) of i.p.l. carrageenan plus s.c. vehicle group were upregulated 24 h after carrageenan injection. Carrageenan-induced upregulation of OX-42 (Figure 5C and iNOS (Figure 5I) were significantly inhibited by s.c. sinularin (80 mg/kg). Quantification results of OX-42 (Figure 5J), GFAP (Figure 5K), and iNOS (Figure 5L supported that inhibition of carrageenan-induced upregulation of spinal neuroinflammatory markers are consistent with the antinociceptive effects of sinularin. To explore the possible effects of sinularin on carrageenan-induced peripheral inflammatory responses, we then focused on the site of rat’s inflamed paw by histopathological examination and immunohistochemistry assay.

**Figure 6:**
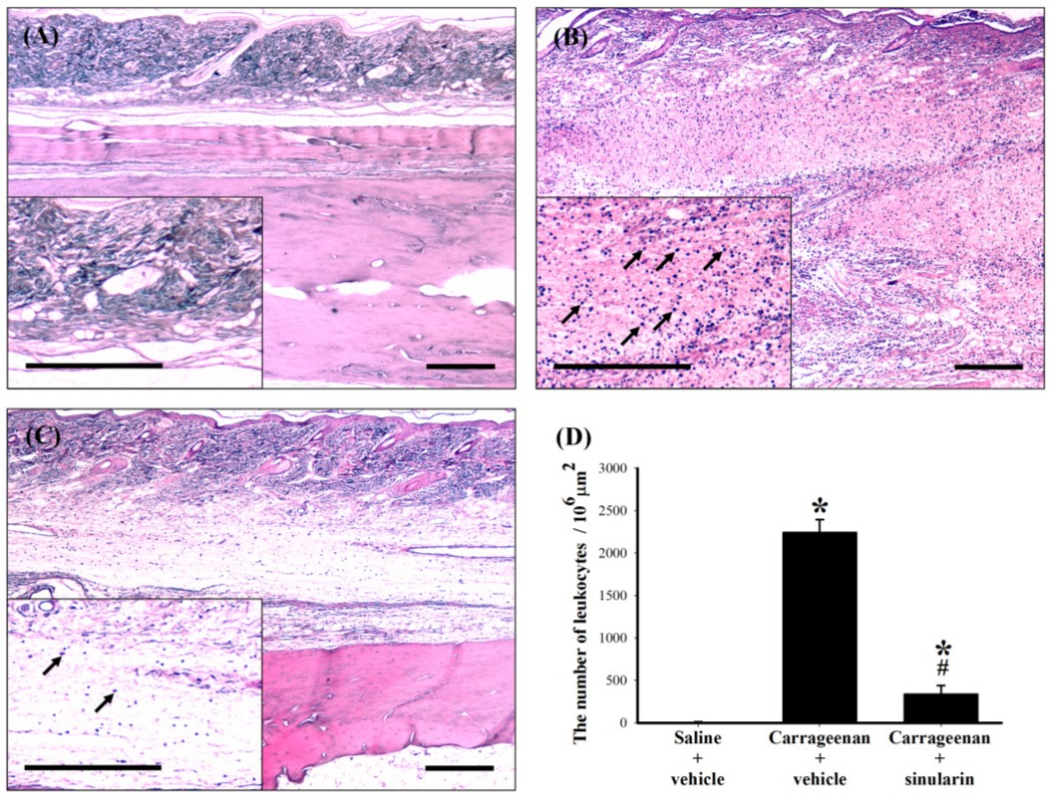
Inhibitive effect of subcutaneous (s.c.) sinularin on intraplantar (i.p.l.) carrageenan-induced leukocyte infiltration in paw tissue. Paw sections (2 μm) at 24 h after i.p.l. saline or carrageenan injection were from (**A**) i.p.l. saline plus s.c. vehicle (dimethyl sulfoxide [DMSO]), (**B**) i.p.l. carrageenan plus s.c. vehicle, and (**C**) i.p.l. carrageenan plus s.c. sinularin (80 mg/kg) groups. (**D**) Quantification of the number of leukocytes (arrows) in the paw tissue, and each bar represents the mean ± standard error of the mean (SEM) of 6 rats per group. S.c. sinularin significantly reduced carrageenan-induced leukocyte infiltration. Scale bars: 300 μm for all images (A–C). * *P* < 0.05 compared with the i.p.l. saline plus s.c. vehicle group; ^#^ *P* < 0.05 compared with the i.p.l. carrageenan plus s.c. vehicle group.

### 2.5. Effect of Systemic Injection of Sinularin on the Carrageenan-Induced Tissue Inflammatory Responses and TGF-β1 Protein Expression in Paws

**Figure 7:**
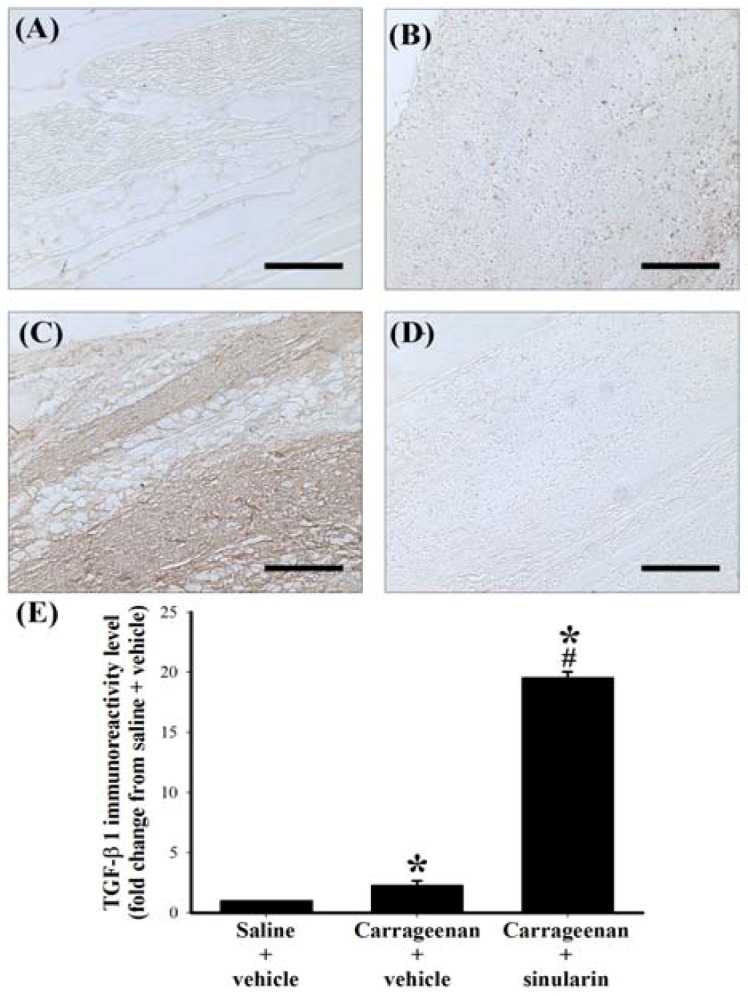
Upregulatory effect of subcutaneous (s.c.) sinularin on intraplantar (i.p.l.) carrageenan-induced transforming growth factor-β1 (TGF-β1) protein expression in paw tissue. Paw sections (2 μm) at 24 h after i.p.l. saline or carrageenan injection were from i.p.l. saline plus s.c. vehicle (dimethyl sulfoxide [DMSO]) (**A**), i.p.l. carrageenan plus s.c. vehicle (**B**, **D**), and i.p.l. carrageenan plus s.c. sinularin (80 mg/kg) (**C**) groups. Immunostaining images show cells labeled with TGF-β1 (**A**–**C**) in the spinal cord, and sample from i.p.l. carrageenan plus s.c. vehicle group incubated without primary antibody for TGF-β1 is presented as a negative control (**D**) and showed no specific staining. Quantification of TGF-β1 immunoreactivity (**E**) in paw tissue, and each bar represents the mean ± standard error of the mean (SEM) of 6 rats per group. S.c. sinularin significantly upregulated carrageenan-induced TGF-β1 immunoreactivity of the paw tissue. Scale bars: 200 μm for all images (A–D). * *P *< 0.05 compared with the i.p.l. saline plus s.c. vehicle group; ^#^ *P* < 0.05 compared with the i.p.l. carrageenan plus s.c. vehicle group.

Histopathological examination was conducted to evaluate tissue inflammatory responses. Biopsies of paws were taken from the following groups: i.p.l. saline plus s.c. vehicle, i.p.l. carrageenan plus s.c. vehicle, and i.p.l. carrageenan plus s.c. sinularin (80 mg/kg) groups at 24 h after i.p.l. carrageenan injection. No inflammation or tissue destruction was observed in the paws of i.p.l. saline plus s.c. vehicle-treated rats (Figure 6A In contrast, as previously reported [1], we observed that i.p.l. carrageenan caused infiltrating cells (leukocytes) populated enlarged cavities resulting from tissue erosion (Figure 6B). Treatment with sinularin (80 mg/kg) clearly inhibited carrageenan-induced leukocyte infiltration (Figure 6C,D). To detect TGF-β1 protein expression in paws, we used paw sections at 24 h after i.p.l. saline or carrageenan injection from i.p.l. saline plus s.c. vehicle, i.p.l. carrageenan plus s.c. vehicle, and i.p.l. carrageenan plus s.c. sinularin (80 mg/kg) groups. At 24 h after i.p.l. carrageenan injection, we found that numerous TGF-β1 immunoreactive cells in the paw tissue (Figure 7B). Administration of sinularin (80 mg/kg) 1 h prior to the injection of carrageenan markedly increased TGF-β1 immunoreactivity in paws (Figure 7C,E).

## 3. Discussion

### 3.1. Summary

In the present study, we found that sinularin significantly inhibits upregulation of the proinflammatory proteins iNOS and COX-2 and upregulates production of the anti-inflammatory protein TGF-β in LPS-stimulated murine macrophage RAW 264.7 cells. Next, we found that s.c. administration of sinularin (80 mg/kg) 1 h before carrageenan injection significantly inhibits carrageenan-induced nociceptive behaviors as well as thermal hyperalgesia, mechanical allodynia, cold allodynia, and hindpaw weight-bearing deficits. Immunohistofluorescence analyses revealed that s.c. sinularin (80 mg/kg) also significantly inhibits carrageenan-induced spinal neuroinflammation, upregulation of microglial and astrocyte immunohistochemical activation markers (OX-42 and GFAP) and iNOS in the dorsal horn of the lumbar spinal cord. Moreover, s.c. sinularin (80 mg/kg) inhibit carrageenan-induced tissue inflammatory responses, redness and edema of the paw, and leukocyte infiltration. Immunohistochemical observations suggested the ability of s.c. sinularin (80 mg/kg) to up-regulate production of TGF-β1 in carrageenan-induced inflamed paw tissue. These present results demonstrate that the marine-derived compound sinularin has anti-inflammatory and analgesic properties in *in vitro *and *in vivo *inflammatory models, respectively.

### 3.2. Analgesic Effects of s.c. Sinularin at Behavioral and Spinal Level

In rat models of intraplantar carrageenan-induced acute inflammatory pain, common carrageenan dosages are between 1% and 2% in a volume of 100 μL [13,14,25,27]. We selected 1.5% carrageenan for the present study based on our previous practical experience [1]. Central sensitization of the spinal cord dorsal horn contributes to hypersensitive pain behaviors of inflammatory pain such as hyperalgesia (thermal hyperalgesia), allodynia (mechanical allodynia and cold allodynia), and spontaneous pain (weight-bearing deficits) [28]. We also confirmed these nociceptive behaviors in a rat model of intraplantar carrageenan, which peaked at 3–6 h and persisted for 24 h after i.p.l. carrageenan injection. Spinal neuroinflammation, primarily characterized by activation of microglia and astrocytes and overexpression of proinflammatory mediators (such as iNOS) in the CNS [3,29], may accelerate central sensitization and result in the development and maintenance of pain [30,31]. Upregulation of immunoreactivity of spinal dorsal horn OX-42 (microglial marker) and GFAP (astrocyte marker) indicate elevated nociceptive states [2,3,15,16,17,18,19,20,21,22]; however, little information is available regarding acute inflammatory pain models of intraplantar carrageenan at common dosages. Only one report showed that intraplantar injection of carrageenan (100 μL, 2%) can upregulate OX-42 and GFAP in the ipsilateral spinal cord dorsal horn of rats 4 h after carrageenan injection, but Xu *et al*. did not directly mention or measure this phenomenon [27]. Our immunohistofluorescence analyses clearly showed carrageenan-induced upregulation of OX-42 and GFAP immunoreactivity in the ipsilateral dorsal region of the lumbar spine at 24 h after carrageenan injection. Additionally, similar to results observed in mice [32,33] but not in rats, we observed upregulation of spinal iNOS immunoreactivity in 24 h after carrageenan injection. In summary, upregulation of spinal OX-42, GFAP, and iNOS immunoreactivity may reflect the level of nociception in carrageenan-induced inflammatory pain in rats. We found that s.c. sinularin significantly inhibited carrageenan-induced spinal neuroinflammation, including upregulation of spinal OX-42, GFAP, and iNOS immunoreactivity, 24 h after carrageenan injection. Thus, based on the results of inhibition of elevated nociceptive states indicators (behavioral testing and spinal immunohistofluorescence), we concluded sinularin attenuates nociceptive sensitization in inflammatory pain rats.

### 3.3. Potential Analgesic Mechanisms of Sinularin

After tissue injury, production of inflammatory mediators causes migration of leukocytes into local inflamed tissue, amplifying the inflammatory response [34]. Systemic administration of the leukocyte inhibitor colchicine suppressed carrageenan-induced paw edema [12,35]; moreover, specific inhibition of leukocyte migration reduced carrageenan-induced mechanical hypernociception by systemic fucoidin [36]. We also found that s.c. sinularin inhibited carrageenan-induced paw edema and nociceptive behaviors accompanied leukocyte infiltration 24 h after carrageenan injection. Therefore, the beneficial effects of sinularin may partially result from its ability to lower the level of tissue inflammation, such as reducing swelling responses and inhibiting leukocyte infiltration. In addition to inhibiting LPS-induced iNOS and COX-2, sinularin also resulted in up-regulation of TGF-β, generally known as the anti-inflammatory cytokine under certain pathological conditions, in LPS-stimulated macrophages. Several previous studies have shown that TGF-β1 has anti-inflammatory effects in LPS-stimulated macrophage cells [37,38,39]. Although the carrageenan-injected paw is a well-characterized inflammatory pain model, only 3 studies have examined TGF-β1 expression in serum [40,41,42], and there are no reports regarding local TGF-β1 expression at the inflammation site in the paw. Compared with decreased serum TGF-β1 [41,42], we observed increased TGF-β1 immunoreactivity in carrageenan-induced inflamed paw tissue. Systemic diclofenac, a nonsteroidal anti-inflammatory drug, prevented a peripheral inflammation-induced decrease of serum TGF-β1 [41]. We also observed that s.c. sinularin (80 mg/kg) may up-regulate TGF-β1 immunoreactivity in carrageenan-induced inflamed paw tissue compared with the saline plus vehicle and carrageenan plus vehicle groups. Indeed, systemic TGF-β1 was shown to have protective functions involving anti-inflammatory effects in collagen-induced arthritis in mice [43]. The analgesic effects of sinularin have been associated with both inhibiting leukocyte infiltration and upregulating TGF-β1, which participate in lowering the level of local tissue inflammation.

### 3.4. Advantages and Future Studies of Sinularin

Based on the present study, there are three advantages of sinularin: low toxicity, no steroid or opioid backbone, and an adequate supply of the compound. Although we did not observe contralateral inflammatory responses in the present experiment, there were no obvious external behavior side effects of carrageenan and sinularin treatment in rats (20 and 80 mg/kg), which is consistent with previous *in vitro* and *in vivo* reports [7,8]. Because the chemical structure of sinularin does not contain a steroid or opioid backbone but was shown to exhibit anti-inflammatory and analgesic activity in the present study, this is important and valuable feature would favor drug development. Sinularin is a major compound in the soft coral genus *Sinularia* [44], and there is thus an adequate supply of compound. Supply often limits development opportunities for *in vivo* bioactivity studies of other minor marine-derived compounds. Moreover, the soft coral genus *Sinularia* has been successfully cultured in the National Museum of Marine Biology and Aquarium (NMMBA), Taiwan [45]. Our present study highlights 2 future studies, which are described below. First, the 80 mg/kg dose of sinularin relieved carrageenan-induced nociceptive behaviors of inflammatory pain, although sinularin dose used in the present study appears to be high; thus, the structure of sinularin should be modified in order to improve its anti-inflammatory activity and analgesic properties followed by additional structure and activity relationship (SAR) studies. Second, we only observed the ability of s.c. sinularin to up-regulate TGF-β1 production in inflamed paw tissue, but the detailed mechanism regarding the modulation effects of sinularin on the TGF-β1 pathway require further investigation. Additionally, we showed that TGF-β1 upregulation may be an *in vitro* or *in vivo* indicator for searching for natural compounds with analgesic activity. The marine-derived compound sinularin was shown to have anti-inflammatory activity three decades ago [7], and no new studies have explored other bioactivities of sinularin. Thus, we hope that the present study involving sinularin will stimulate further study in the field of marine drugs.

## 4. Methods and Materials

### 4.1. Chemicals

The marine natural compound sinularin (5,15-dioxatricyclo[12.3.1.0(4,6)]octadec-9-en-16-one; Figure 1) was isolated from the soft coral *Sinularia querciformis* collected from the Dongsha Islands near Taiwan. We purchased DMSO and carrageenan lambda from Sigma Co., Ltd. (St. Louis, MO, USA) and indomethacin from Cayman Chemical Co. (Ann Arbor, MI, USA).

### 4.2. *In Vitro* Anti-Inflammatory Assay

We performed an *in vitro* anti-inflammatory assay according to the method described in our previous study [1,4,10,11]. We obtained murine RAW 264.7 macrophages from the American Type Culture Collection (ATCC, No TIB-71). After incubating macrophage cells for 16 h in Dulbecco’s modified Eagle medium (DMEM; contains 10% heat-inactivated fetal-bovine serum (FBS), 1 mM pyruvate, 2 mM glutamine, 4.5 g/L glucose, 50 μg/mL streptomycin, and 50 U/mL penicillin under standard conditions in a 37 °C humidified atmosphere of 5% CO_2_: 95% air) containing only LPS (0.01 μg/mL; Sigma L2654), an *in vitro* inflammatory state was induced in the macrophages. For the anti-inflammatory activity assay, sinularin (0.1, 1, 10, or 20 μM) was added to the cells 5 min prior to the LPS challenge. Next, cells were washed with ice-cold phosphate-buffered saline (PBS), lysed with ice-cold lysis buffer (1 μg/mL aprotinin, 150 mM NaCl, 100 μg/mL phenylmethylsulfonyl fluoride, 50 mM Tris, pH 7.5, 1% Triton X-100), and centrifuged for 30 min at 20,000 × *g* at 4 °C. The supernatant was decanted from the pellet and retained for Western blot analysis to detect the presence of iNOS, COX-2, and TGF-β. Protein concentrations of whole cell lysates were determined with the DC protein assay kit (Bio-Rad, Hercules, CA, USA) using a method modified from Lowry *et al.* [46].

### 4.3. Western Blot Analysis to Determine the Presence of iNOS, COX-2, and TGF-β

We performed western blot analysis using our published method [2]. An equal volume of sample buffer (0.1% bromophenol blue, 10% glycerol, 2% 2-mercaptoethanol, 2% sodium dodecyl sulfate (SDS), and 50 mM Tris–HCl, pH 7.2) was added to whole cell lysates (supernatant), which was then electrophoresed on a tricine SDS-polyacrylamide gel at 150 V for 90 min. Proteins were transferred from the gel to a polyvinylidene difluoride (PVDF) membrane (Immobilon-P; Millipore Corporation, Billerica, MA, USA; 0.45-μM pore size) in transfer buffer (380 mM glycine, 20% methanol, 1% SDS, and 50 mM Tris–HCl) at 125 mA overnight at 4 °C. The PVDF membrane was blocked for 1 h at room temperature with 5% non-fat dry milk in Tris-buffered saline (TTBS; 137 mM NaCl, 20 mM Tris–HCl, 0.1% Tween 20, pH 7.4), and then incubated for 180 min with antibodies against iNOS (1:1000 dilution; BD Pharmingen, San Diego, CA, USA; catalog no. 6103322; polyclonal antibody), COX-2 (1:1000 dilution; Cayman Chemical, Ann Arbor, MI, USA; catalog no. 160106; polyclonal antibody), or TGF-β (1:1000 dilution; Abcam, Cambridge, UK; catalog no. ab66043; polyclonal antibody) proteins at room temperature. The iNOS, COX-2, and TGF-β antibodies were used to identify bands at approximately 135, 70, and 13 kDa, respectively. Immunoreactive bands were visualized using enhanced chemiluminescence (ECL kit; Millipore). Bands were visualized and photos were acquired using the UVP BioChemi imaging system (UVP LLC, Upland, CA, USA) and relative densitometric quantification of immunoreactive bands was performed using LabWorks 4.0 software (UVP LLC). PVDF membranes were re-probed using an anti-β-actin antibody as a loading control (monoclonal mouse antibody, 1:2500 dilution; catalog no., A5441; Sigma). Relative variations between the bands of sinularin-treated samples and LPS-treated samples were calculated using the same images.

### 4.4. Carrageenan-Induced Paw Edema and Behavioral Testing

Male Wistar rats (285–300 g) with free access to food and water were housed within a temperature-controlled (22 ± 1 °C) room with a 12-h/12-h light/dark schedule. In accordance with the Guiding Principles in the Care and Use of Animals of the American Physiology Society, experiments involving rats were approved by the National Sun Yat-sen University and Use Committee. Every effort in experimental design and execution was made in order to minimize the number of rats used and to minimize their suffering. Paw edema was induced by i.p.l. injection of 1.5% sterile carrageenan lambda in 100 μL of saline into the right hind paw [47]. Sinularin or indomethacin was dissolved in DMSO and delivered in a volume of 300 μL in preparation for s.c. administration to rats. Vehicle (DMSO; 300 μL), sinularin, and indomethacin were administrated 1 h before i.p.l. carrageenan injection.

#### 4.4.1. Paw Edema

Paws were marked in order to assist in replication of the positioning in the measurement chamber using a method described in our previous report [1]. Increases in paw volume were measured using a paw volume meter (plethysmometer; Singa Technology Corporation) and calculated by deducting the basal paw volume (before i.p.l. carrageenan injection) from the paw volume measured at each time point (after i.p.l. carrageenan injection). To simplify the data analysis for statistical purposes, the AUC of the edematous effect-time curve for the plot of paw edema versus time was computed using the trapezoidal method [48] from 0 to 24 h after i.p.l. carrageenan plus s.c. administration of vehicle, sinularin, or indomethacin.

#### 4.4.2. Thermal Hyperalgesia

To test thermal hyperalgesia, we used an IITC analgesiometer (IITC Inc., Woodland Hills, CA, USA) as described previously by Hargreaves *et al*. [49] and our previous study [1]. Briefly, rats were placed in compartments of clear plastic cages on top of an elevated glass plate. A heat stimulus was positioned and directed onto the middle of the plantar surface using a radiant heat source with low-intensity heat (active intensity = 25) until the rat exhibited a positive sign of pain behavior (licking or withdrawal). PWL (in seconds) was measured with a cut-off time of 30 s as the average of 2 measurements per paw.

#### 4.4.3. Mechanical Allodynia

To assess mechanical allodynia, we measured the PWT (in gram) using calibrated von Frey filaments (Stoelting, Wood Dale, IL, USA). Rats were placed in compartments of clear plastic cages on top of an elevated metal mesh floor, permitting easy access to the rats’ paws. A series of von Frey filaments of logarithmically incremental stiffness were applied to the midplantar region of the rat hindpaw from below the mesh floor using Chaplan’s “up–down’’ method involving the use of alternate larger and smaller fibers to determine the closest filament to the threshold of pain response (licking or withdrawal) as described previously by Chaplan *et al*. [50] and by our previous study [3].

#### 4.4.4. Cold Allodynia

We placed the rats in the same apparatus (individual plastic compartments on an elevated metal mesh floor) as that described for the mechanical allodynia test. Pain responses of rats were monitored for 1 min after acetone application (25 μL) at the center of the plantar surface of a hindpaw and were graded according to a 6-point scale, modified from a 4-point scale used in previous studies [51,52]: 5, no response; 4, withdrawal, flick or stamp of the paw more than 2 s after acetone application; 3, quick withdrawal, flick or stamp of the paw in 2 s after acetone application; 2, quick and more violent withdrawal, flick or stamp of the paw within 2 s of acetone application; 1, prolonged withdrawal or repeated flicking of the paw within 2 s of acetone application; 0, repeated flicking of the paw with persistent licking the paw within 2 s of acetone application. Acetone response scores for each rat were then obtained by summing the four individual scores; the minimum possible score was 0 points (repeated flicking and licking of paws on each of the four trials) and the maximum possible score was 20 points (no response to any of the four trials).

#### 4.4.5. Weight-Bearing Deficits

Rats were placed on an incapacitance tester (Singa Technology Corporation, TW) with their hindpaws centered on two force transducers for measuring the weight distribution between the rat’s hind limbs as described in our previous study [53]. Under normal conditions, naïve rats distribute their weight equally between both hind limbs; however, following induction of limb inflammation (such as i.p.l. carrageenan injection), rats redistribute their weight to reduce weight applied to the affected limb [54]. Changes in hind paw weight distribution (in gram) are expressed as the difference between the affected limb (the right hind limb) the normal limb (the left hind limb) measured at the same time point. Two or 3 recordings were taken for each rat to determine the average of measurements at each time point.

### 4.5. Spinal Immunohistofluorescence Assay

Using the spinal immunofluorescence method described in our previous study [2,3], to diminish variations in immunohistochemical procedures, lumbar spinal cord tissues from different groups were mounted on the same OCT block and sectioned together on a cryostat at −30 °C (HM550; Microm, Waldorf, Germany). The 10-μm sections were then incubated with the monoclonal antibody OX-42 (CD11b, microglia marker, 1:200 dilution; Serotec Ltd., Oxford, United Kingdom), GFAP (astrocyte marker, 1:200 dilution, cat. 131-17719; Molecular Probes, Eugene, OR, USA), or anti-iNOS (1:200 dilution) overnight at 4 °C, followed by Alexa Fluor 488-labeled chicken anti-mouse IgG antibody (1:400 dilution; Molecular Probes; catalog no. A-21200; green fluorescence) or DyLight 549-conjugated donkey anti-rabbit IgG (1:400 dilution; Jackson ImmunoResearch Laboratories Inc., West Grove, PA, USA; catalog no. 711-506-152; red fluorescence) for 40 min at room temperature. For immunostaining analysis, stained spinal sections were examined using a Leica DM-6000 CS fluorescence microscope (Leica Instruments Inc., Wetzlar, Germany). All immunofluorescence images of OX-42, GFAP, and iNOS were acquired using a SPOT Xplorer Digital camera (Diagnostic Instruments, Inc., Sterling Heights, MI, USA). Immunofluorescence data were acquired at 100× magnification, then pixel values of the immunoreactive-positive area were counted using Image J software (National Institutes of Health, Bethesda, MD, USA) using three sections per rat, and expressed as a percentage change compared to the i.p.l. saline plus s.c. vehicle group, which were considered to be 100%.

### 4.6. Histopathology and Immunohistochemistry of Paw Tissues

For histopathological examination, we modified a method used in our previous study [1]. After rats were perfused intracardially with 500 mL of cold PBS containing heparin (0.2 U/mL) and 1% sodium nitrite followed by 4% paraformaldehyde in 500 mL of 0.1 M PBS (pH 7.4), paw samples were fixed in 10% neutral buffered formalin, soaked in decalcifying solution for 30 h, and then stocked in 10% formalin prior to following processing. Rat paws were bisected longitudinally, then placed in embedding cassettes for dehydration, clearing, and infiltration by automatic tissue processor (Tissue-Tek, Sakura Finetek Japan Co., Ltd., Japan), embedded into a paraffin block by tissue embedding center (EC780-1; EC780-2, CSA), and cut into 2-μm sections using a rotary microtome (HM340E, Microm). Paw sections were stained with hematoxylin and eosin (H & E) for histopathological examination. For immunohistochemistry, after deparaffinization of the paw sections, endogenous peroxidase of the paw sections was quenched with 0.3% H_2_O_2_ in 60% methanol for 30 min, and then permeabilized using 0.1% Triton X-100 in PBS for 20 min. To minimize nonspecific adsorption, paw sections were incubated with 5% normal goat serum in PBS for 30 min. Paw sections were incubated overnight at 4 °C with anti-TGF-β1 (1:200 dilution; Abcam, Cambridge, UK; catalog no. ab92486; polyclonal antibody) antibody. To detect specific labeling, paw sections were incubated with biotin-conjugated goat anti-rabbit IgG and avidin-biotin-peroxidase complex (Vectastain ABC kit; Vector Laboratories Inc., Burlingame, CA, USA). Finally, paw sections were incubated with 3,3′-diaminobenzidine tetrahydrochloride (DAB) (Vectastain ABC kit; Vector Laboratories) for 5 min. We analyzed all slides of the paw sections for histopathology and immunohistochemistry under a Leica DM-6000 CS microscope (Leica Instruments Inc., Wetzlar, Germany) and a microscope digital camera system (SPOT Idea 5 MP CMOS scientific color digital camera system; Diagnostic Instruments). Immunohistochemical data were acquired at 100× magnification, measured for their individual pixel values of the positive area using MetaMorph Imaging System software (Molecular Devices, Downington, PA, USA) using three sections per rat, and expressed as a percentage change compared to the i.p.l. saline plus s.c. vehicle group, which were considered to be 100%.

### 4.7. Data and Statistical Analysis

All data are shown as means ± standard error of the mean (SEM). For statistical analysis, differences between groups of macrophage cell lines or rats were calculated using one-way analysis of variance (ANOVA), followed by the Student-Newman–Keuls *post hoc* test. We defined statistical significance as *P* < 0.05.

## 5. Conclusions

The *in vitro* anti-inflammatory activity of sinularin was associated with inhibition of iNOS and COX-2 and upregulation of TGF-β. Further, the analgesic properties of sinularin in *in vivo *experiments have not been previously reported. The present study shows that systemic sinularin exerts its analgesic effects at the behavioral and spinal levels, which are associated with both inhibition of leukocyte infiltration and upregulation of TGF-β1.
